# Antimicrobial resistance and stewardshipin clinical practice: A cross-sectional study of healthcare professionals in Mogadishu, Somalia

**DOI:** 10.1371/journal.pgph.0006032

**Published:** 2026-06-18

**Authors:** Bashiru Garba, Muna Sheikh Mohamed, Musab Mohamud Ahmed, Omar Abdullahi Abdi, Muna Ibrahim Hassan, Mohamed Ali Ibrahim, Najma Hashim Sayid Macani, Yushau Umar, Fartun Abdullahi Hassan Orey

**Affiliations:** 1 SIMAD Institute for Global Health, SIMAD University Mogadishu, Mogadishu, Somalia; 2 Faculty of Medicine and Health Sciences, SIMAD University Mogadishu, Mogadishu, Somalia; 3 National Veterinary Research Institute Vom, Jos, Plateau State, Nigeria; 4 Department of Pediatrics and Child Health, Dr Sumait Hospital, SIMAD University Mogadishu, Mogadishu, Somalia; University of Oslo Faculty of Medicine: Universitetet i Oslo Det medisinske fakultet, NORWAY

## Abstract

Antimicrobial resistance is an escalating threat that undermines the effective treatment of common infections and increases morbidity, mortality, and healthcare costs. In fragile health systems such as Somalia’s, limited laboratory capacity, variable prescribing practices, and easy access to antibiotics can accelerate inappropriate use and resistance. This study assessed attitudes, general knowledge about antibiotics, and awareness of AMR and antimicrobial stewardship (AMS) among healthcare professionals in Mogadishu and examined associated socio‑demographic and professional factors. A cross‑sectional survey was conducted among 426 healthcare professionals from seven hospitals in Mogadishu. Participants included physicians, nurses and midwives, pharmacists, laboratory technicians, and other patient-care professionals, including radiology and allied clinical staff. Data were collected using a structured, self‑administered questionnaire covering socio‑demographic characteristics, attitudes towards actions to combat AMR, general knowledge about antibiotics, and awareness of AMR and AMS. Likert‑scale responses (1–5) were summed to generate composite scores. Descriptive statistics summarized scores, Spearman correlations assessed relationships between domains, and linear regression identified predictors of attitudes, knowledge, and awareness. Mean (SD) scores were 58.93 (7.73)/74 for attitudes (78.6%), 38.10 (6.90)/55 for knowledge (69.3%), and 36.07 (5.31)/49 for awareness (72.1%). Knowledge correlated with attitudes (ρ = 0.46, p < 0.001) and awareness (ρ = 0.53, p < 0.001); awareness also correlated with attitudes (ρ = 0.40, p < 0.001). While most respondents endorsed stewardship‑aligned actions, many held misconceptions, including using antibiotics for all infections, stopping treatment when feeling better, and reusing leftover antibiotics. Holding a master’s degree predicted higher attitude scores; being 25–30 years old and working in specific hospitals predicted higher knowledge. Having 6–10 years of practice was negatively associated with knowledge and awareness. Healthcare professionals in Mogadishu show strong pro‑stewardship attitudes and reasonable awareness but important knowledge gaps, especially among mid‑career staff, highlighting priority targets for context-adapted AMS interventions, particularly by years of experience, education level, and facility context.

## Introduction

Antimicrobial resistance (AMR) is a major global health threat, with the greatest burden in low‑ and middle‑income countries where fragile health systems and weak regulation drive inappropriate antimicrobial use [1]. In such settings, widespread empirical prescribing, over‑the‑counter antibiotic sales, and limited diagnostic capacity accelerate the emergence and spread of resistant pathogens and undermine routine clinical care [2–5]. Constrained laboratory services, poorly regulated medicine markets, and gaps in prescribers’ and dispensers’ knowledge and practices further exacerbate the problem [4]. Healthcare professionals are central to this dynamic, as their knowledge, attitudes, and everyday decisions directly shape antibiotic consumption and the implementation of stewardship principles [6,7].

These challenges are particularly acute in many LMICs, where limited diagnostics, non-prescription access to antibiotics, weak regulatory frameworks, shortages of trained personnel, and poor infection prevention and control (IPC) infrastructure fuel inappropriate antimicrobial use [8,9]. Decades of conflict, political instability, and recurrent disasters have disrupted health services, constrained public financing, and fragmented governance, leaving much of the population reliant on under‑resourced and poorly regulated providers. Recent analyses suggest that Somalia is among the countries with the highest AMR-attributable mortality globally, with thousands of deaths annually linked to resistant infections, in the context of very low Global Health Security Index scores and limited capacity to detect and respond to AMR threats [10,11]. Weak regulatory oversight, widespread access to antimicrobials without prescriptions, and inadequate IPC capacity in many facilities further fuel misuse across public, private, and informal care [10,11].

In Mogadishu, the capital and main referral hub, rapid urbanisation, recurrent displacement, and a mixed landscape of public, private and charitable hospitals complicate efforts to govern antimicrobial use and standardise clinical practice. Evidence from Somali hospitals describes high levels of empirical prescribing, frequent use of broad‑spectrum agents, limited access to microbiology, and poor IPC practices, all favouring the selection and transmission of multidrug‑resistant organisms [12]. National initiatives, including a National Action Plan on AMR and early efforts to establish surveillance and stewardship structures, signal growing recognition of the problem, but implementation remains uneven and under‑resourced, especially in crisis‑affected urban settings such as Mogadishu [13].

Across Africa and other low‑resource settings, surveys among healthcare workers consistently document important gaps in AMR and antimicrobial stewardship (AMS) knowledge and awareness, alongside generally positive attitudes towards responsible antibiotic use [6,7]. Professional cadre, training level, and experience are associated with substantial variation in AMR‑related competencies, highlighting the need for locally tailored stewardship training and system‑level support to translate favourable attitudes into improved prescribing and IPC practice. However, there are very limited data from Somalia, particularly on how frontline staff in Mogadishu’s diverse and weakly regulated hospital sector understand AMR and stewardship or how their sociodemographic and professional characteristics shape these domains.

Against this backdrop, this cross‑sectional study aimed to assess attitudes, general knowledge about antibiotics, and awareness of AMR and stewardship among healthcare professionals working in major hospitals in Mogadishu, Somalia. Specifically, the objectives were to describe overall and item‑level patterns in these domains and to identify sociodemographic and professional factors associated with attitudes, knowledge, and awareness in a protracted‑crisis, disaster‑prone, and weakly regulated health system context.

## Methods

### Study design and setting

This was a cross‑sectional survey conducted among healthcare professionals working in Mogadishu, the capital of Somalia. The study was implemented in six of the 20 districts in Mogadishu, including Hodan, Wadajir, Yaqshid, Warta Nabada, Darussalam, and Howlwadaag, each represented by at least one hospital facility. These districts were randomly selected, and the resulting sample happened to include a mix of public and private facilities that provide primary, secondary, and tertiary care in an urban, crisis‑affected setting. However, data collection was ultimately conducted in seven hospitals located across these six districts.

The survey was conducted using consecutive/available sampling of eligible HCWs within participating hospitals. All HCWs who were permanent staff or visiting consultants and directly involved in patient care or antimicrobial use at the time of data collection were eligible for inclusion, while those in purely administrative positions with no clinical role were excluded.

### Study population and sample size

The target population comprised medical doctors, nurses, midwives, pharmacists, laboratory technicians, radiology personnel, public health practitioners, and other allied clinical staff (including anesthesiologists, dentists, biomedical engineers, optometrists, etc.) working in the selected facilities in Mogadishu. The sample size was determined using the standard formula for prevalence studies, which is widely applied in health research where the primary outcome is a proportion or prevalence [14]. Based on an estimated population of approximately 6,000 healthcare workers in Somalia [15], a conservative prevalence estimate of 50% was assumed to maximize sample size, with a 95% confidence level and a margin of error of 5% [16]. This yielded a minimum required sample size of 375 participants. To account for potential non-response and incomplete questionnaires, the target sample size was increased by approximately 10% [17].

The initial sampling strategy aimed to distribute participants evenly across six key districts in Mogadishu, with an estimated target of approximately 66 healthcare workers per district, reflecting an effort to achieve broad geographic representation within the city. However, during field implementation, access and administrative approvals could not be secured uniformly across all districts. Consequently, the sampling approach was adapted to a facility-based strategy, whereby data collection was conducted in hospitals where formal approval and access were granted, irrespective of district location, a pragmatic approach commonly adopted in fragile and resource-constrained settings. This adaptive strategy has been shown to be appropriate in humanitarian and conflict-affected contexts, where rigid sampling frames are often not feasible due to insecurity, governance constraints, and variable institutional access [18].

Prior to data collection, a pre-testing of the questionnaire was conducted among healthcare workers in a different hospital in Mogadishu. The questionnaire was pre-tested to assess clarity, relevance, and internal consistency and to ensure alignment with the study objectives. A reliability score of 0.85 (Cronbach’s alpha) was obtained from the pre-test analysis, confirming the instrument’s internal consistency [19].

During the initial phase of data collection, responses were obtained from Madina Hospital, Mogadishu Specialist Hospital, Horyaal Hospital, Dr. Sumait Hospital, and Welcare Hospital. Data collection was then extended to Demartino Public Hospital and Shaafi Hospital, with additional recruitment at Dr. Sumait Hospital, resulting in a final sample of 426 participants. This is done to improve balance across sites, following principles of maximizing participation where access was granted [20].

Through these efforts, a total of 426 healthcare professionals were ultimately enrolled in the study. Although this final sample exceeded the initially calculated target of 413 participants, inclusion of all completed questionnaires was retained, as larger sample sizes increase the precision of estimates and statistical power without introducing bias when eligibility criteria remain unchanged [21].

### Data collection procedure

Data were collected from 20/06/2025 to 29/09/2025 using a modified questionnaire (S1 File) adapted from a previous study, with minor contextual modifications for the present study (Kpokiri et al. 2022) [22]. The survey instrument comprised four sections: (i) socio-demographic characteristics of healthcare workers (HCWs), (ii) general knowledge regarding antimicrobials and their use, (iii) awareness of AMR and AMS, and (iv) attitudes towards actions to combat AMR. Responses to Likert-scale items were coded on a five-point scale ranging from strongly disagree to strongly agree, a method commonly employed in knowledge and attitude surveys. Negatively worded items were reverse coded to ensure consistency in score directionality. For presentation purposes, responses of “agree” and “strongly agree” were combined and reported as “agreed,” while “disagree” and “strongly disagree” were combined and reported as “disagreed,” consistent with reporting approaches used in antimicrobial stewardship and AMR perception studies [6,7].

Knowledge of antimicrobial use was assessed using 11 items, awareness using 10 items, and attitude using 13 items, with score ranges defined according to the structure of the questionnaire. Composite scores were treated as continuous variables; higher scores indicated more favorable attitudes, better knowledge, or greater awareness. Internal consistency was assessed for each domain using Cronbach’s alpha (α = 0.83, α = 0.82, & α = 0.80). Principal component analysis (PCA) was conducted to examine the scale dimensionality, which indicated a dominant first component (eigenvalues = 4.33, 3.7, 3.5) across all outcome variables, supporting one-dimensionality. The composite score of the outcome variables was therefore treated as a continuous variable in subsequent analyses [23,24]. Thresholds at 80% were explored but not used for primary analyses, which focused on mean scores and regression models [25].

Participants scoring below this threshold were categorized as having poor knowledge, low awareness, or poor attitudes regarding antimicrobial use, antimicrobial resistance, and antimicrobial stewardship, consistent with prior KAP-based analytical frameworks. The profession variable in the demographic domain was collapsed into five groups (physicians, nurses/midwives, laboratory technicians, pharmacists, and other professions) to reduce sparsity and improve model stability and interpretability in descriptive and bivariate analyses.

### Ethical approval

Before conducting the study, we obtained ethical approval from the Faculty of Medicine and Health Sciences Ethics Committee with an approval number of REF:0003/SU/SIGHt-R027/11/06/25. All the participants were informed about the purpose of the study. Participation was voluntary after providing informed and written consent.

### Data analysis

Data were analysed using Stata 19.5. Descriptive statistics (means, standard deviations, frequencies, and percentages) summarised the data, and independent t‑tests and one‑way ANOVA compared mean scores across predictor groups, with Bonferroni post‑hoc tests where appropriate. Variables with p < 0.20 entered multivariable linear regression models to identify predictors of attitudes, knowledge, and awareness. Multicollinearity was assessed using variance inflation factors (VIF), with all values below 5 (range: 2.85-3.24), indicating no evidence of problematic collinearity. Residual diagnostics, including histograms and normal probability plots, suggested approximate normality, and plots of residuals versus fitted values indicated no substantial heteroscedasticity. Model fitting was compared using the Akaike information criterion, and the best model with minimal AIC was selected in this study. Where alternative model specifications were compared, the more parsimonious model with the lower AIC was retained; accordingly, profession was excluded from the final multivariable models after comparison with models that included the collapsed profession categories.

## Results

### Characteristics of the study population

A total of 426 healthcare professionals from seven Mogadishu hospitals were included in the analysis. The mean age was 26.8 years (SD 7.2; range 18–80), and most were younger than 30 years (42.5% < 25 years; 42.7% 25–30 years). Females comprised 40.9% of participants, and most held a bachelor’s degree (82.6%), with 3.1% holding a diploma/HND, 10.8% a master’s degree, and 3.5% a PhD. When professions were collapsed into broader analytical categories, nurses and midwives constituted the largest group, followed by physicians and laboratory technicians, while pharmacists and other professions accounted for smaller proportions of the sample. Over half had 1–5 years of practice (55.6%), and nearly one quarter had less than 1 year of experience, indicating a predominantly early‑career workforce (Table 1).

**Table 1:** Socio‑demographic and professional characteristics of healthcare professionals.

Variables	Study sample (n = 426)
**Age**	
Less than 25 years	181 (42.49%)
25-30 years	182 (42.72%)
31-40 years	42 (9.86%)
More than 40 years	21 (4.93%)
Min. - Max.	18.0 - 80.0
Mean ± SD	26.83 ± 7.19
**Sex**	
Male	252 (59.15%)
Female	174 (40.85%)
**Highest educational qualification**	
Diploma/HND	13 (3.05%)
Bachelor’s degree	352 (82.63%)
Master’s degree	46 (10.80%)
PhD	15 (3.52%)
**Profession**	
Other professions	19 (4.46%)
Pharmacies	12 (2.82%)
Laboratory Technician	49 (11.50%)
Physicians	143 (33.57%)
Nurses/Midwives	203 (47.65%)
**Years of practice**	
Less than 1 year	101 (23.71%)
1-5 years	237 (55.63%)
6-10 years	55 (12.91%)
More than 10 years	33 (7.75%)
**Hospital**	
Demartino Public Hospital	65 (15.26%)
Dr Sumait Hospital	62 (14.55%)
Horyaal Hospital	69 (16.20%)
Madina Hospital	66 (15.49%)
Mogadishu Specialist Hospital	66 (15.49%)
Shaafi Hospital	80 (18.79%)
Welcare Hospital	18 (4.23%)

### Attitudes, knowledge and awareness scores

The overall mean score for attitudes towards actions to combat antimicrobial resistance was 58.93 (SD 7.73) out of a possible 74, corresponding to a mean percentage score of 78.57% (Table 2). On the other hand, the mean general knowledge score about antibiotics was 38.10 (SD 6.90) out of 55 (mean percentage 69.27%), while the mean awareness score for antimicrobial resistance and stewardship was 36.07 (SD 5.31) out of 49 (mean percentage 72.13%) (Table 2).

**Table 2:** Mean scores for attitudes, general knowledge about antibiotics, and awareness of antimicrobial resistance and stewardship.

Total scores	Study sample (n = 426)
Attitudes on actions to combat antimicrobial resistance	
Min. - Max.	32 - 74
Mean ± SD	58.93 ± 7.73
Mean %	78.57%
General knowledge about antibiotics	
Min. - Max.	19 - 55
Mean ± SD	38.10 ± 6.90
Mean %	69.27%
Awareness of antimicrobial resistance and stewardship	
Min. - Max.	17 - 49
Mean ± SD	36.07 ± 5.31
Mean %	72.13%

Note: Mean % was calculated as (mean score ÷ maximum possible score) × 100 and reflects the average level of agreement/endorsement on the scale.

**Fig 1:**
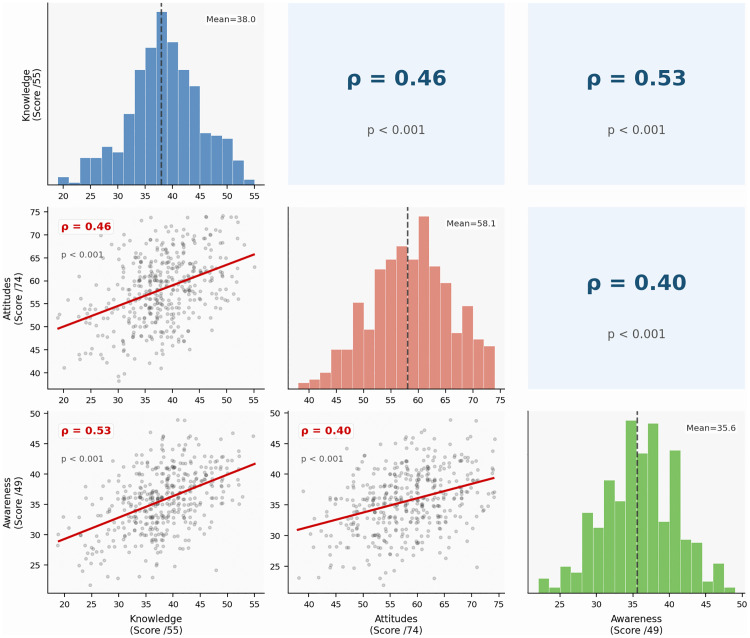
Spearman correlation scatter plot matrix showing pairwise relationships between knowledge, attitudes, and awareness scores (n = 426). Upper panels show Spearman rho (ρ) values; lower panels show scatter plots with linear regression lines; diagonal panels show score distributions. All correlations are statistically significant at p < 0.001.

There were strong, statistically significant positive correlations between the three domains: knowledge correlated with attitudes (ρ ≈ 0.46, p < 0.001) and awareness (ρ ≈ 0.53, p < 0.001), and awareness also correlated with attitudes (ρ ≈ 0.40, p < 0.001), suggesting that respondents with better knowledge tended to report more positive attitudes and higher awareness of antimicrobial resistance and stewardship (Table 3 and Fig 1). The scatter plots in Fig 1 visually confirm these associations, with the strongest correlation observed between knowledge and awareness (ρ = 0.53), followed by knowledge and attitudes (ρ = 0.46), and awareness and attitudes (ρ = 0.40). The positive linear trends across all domain pairs indicate that improvements in knowledge are consistently accompanied by higher awareness and more favorable attitudes toward antimicrobial stewardship

**Table 3:** Correlations between attitudes, general knowledge about antibiotics, and awareness of antimicrobial resistance and stewardship.

Variable	Correlation coefficient (ρ)	p-value
Knowledge-Attitudes	0.458422	< 0.001
Knowledge - Awareness	0.530792	< 0.001
Awareness - Attitudes	0.39538	< 0.001

### Item‑level attitudes towards antimicrobial resistance and stewardship

Most participants agreed that antibiotic prescribing should be guided by patients’ clinical condition and, where available, microbiology results (Table 4). They widely recognised key drivers of resistance in their hospitals, including poor infection control, inappropriate prescribing, limited diagnostics, self‑medication and poor hygiene, and many perceived antibiotics to be overused, although perceptions of overuse were more variable than for other items (Table 4). Most also endorsed stewardship‑supportive behaviours, such as consulting experts, using local resistance data, targeting likely pathogens and changing prescriber and patient attitudes to curb unnecessary antibiotic use (Table 4).

**Table 4:** Item‑level attitudes towards actions to combat antimicrobial resistance and stewardship.

Questions	Strongly agree n (%)	Agree n (%)	Neutral n (%)	Disagree n (%)	Strongly disagree n (%)
The patient’s clinical condition influences the decision to start antimicrobial therapy	177 (41.5)	183 (43.0)	41 (9.6)	18 (4.2)	7 (1.6)
Some infections cannot be treated effectively with the current antibiotics available	123 (28.9)	202 (47.4)	52 (12.2)	41 (9.6)	8 (1.9)
There are policies and protocols guiding antibiotic use in this facility	129 (30.3)	186 (43.7)	65 (15.3)	31 (7.3)	15 (3.5)
All prescriptions are based on the hospital’s protocol	141 (33.1)	174 (40.8)	53 (12.4)	46 (10.8)	12 (2.8)
Poor infection control practices cause the spread of antimicrobial resistance	168 (39.4)	168 (39.4)	43 (10.1)	37 (8.7)	10 (2.3)
Consulting with infectious disease experts helps control antimicrobial resistance	187 (43.9)	164 (38.5)	51 (12.0)	14 (3.3)	10 (2.3)
Obtaining local antibiotic resistance profiles helps improve antibiotic use	134 (31.5)	197 (46.2)	46 (10.8)	35 (8.2)	14 (3.3)
Targeting antimicrobial therapy to likely pathogens helps control resistance	139 (32.6)	197 (46.2)	57 (13.4)	23 (5.4)	10 (2.3)
Changing attitudes of prescribers and patients reduces unnecessary antibiotic use	136 (34.3)	170 (39.9)	62 (14.6)	39 (9.2)	9 (2.1)
Microbiological results in symptomatic patients influence the decision to start antibiotics	154 (36.2)	185 (43.4)	52 (12.2)	28 (6.6)	7 (1.6)
Inappropriate prescribing habits contribute to antimicrobial resistance	156 (36.6)	162 (38.0)	60 (14.1)	37 (8.7)	11 (2.6)
Lack of effective diagnostic tools contributes to inappropriate antibiotic use	151 (35.4)	186 (43.7)	52 (12.2)	29 (6.8)	8 (1.9)
Patient self-medication with antibiotics contributes to antimicrobial resistance	199 (46.7)	128 (30.0)	54 (12.7)	33 (7.7)	12 (2.8)
Poor hygiene practices in healthcare settings promote bacterial spread and resistance	191 (44.8)	169 (39.7)	38 (8.9)	21 (4.9)	7 (1.6)
Antibiotics are overprescribed in this facility	98 (23.0)	145 (34.0)	78 (18.3)	85 (20.0)	20 (4.7)

### General knowledge about antibiotics

Many participants held important misconceptions about antibiotic use, including believing that antibiotics are needed for all infections, that treatment can stop once symptoms improve, or that leftover or previously effective antibiotics can be reused without a prescription (Table 5). Although most recognised that frequent antibiotic use reduces effectiveness and should be strictly controlled, these errors-especially around indications, duration and non‑prescription use left the mean knowledge score just below 70%, indicating only partial understanding of key concepts (Table 2 and Table 5).

**Table 5:** Item‑level general knowledge about antibiotics among healthcare professionals.

Questions	Strongly agree n (%)	Agree n (%)	Neutral n (%)	Disagree n (%)	Strongly disagree n (%)
Antibiotics are used in the management of all infections	166 (39.0)	129 (30.3)	36 (8.5)	67 (15.7)	28 (6.6)
Treatment with antibiotics should stop once a patient feels better, especially with expensive antibiotics	117 (27.5)	148 (34.7)	57 (13.4)	73 (17.1)	31 (7.1)
It is acceptable to use leftover antibiotics from a family member or friend if the symptoms are similar	63 (14.8)	137 (32.2)	53 (12.4)	94 (22.1)	79 (18.5)
It is acceptable to buy the same antibiotics without a prescription if they have helped in the past	73 (17.1)	112 (26.3)	50 (11.7)	101 (23.7)	90 (21.2)
Frequent use of antibiotics may decrease treatment efficacy	131 (30.8)	175 (41.1)	57 (13.4)	47 (11.0)	16 (3.8)
Antibiotic use should be strictly controlled	169 (39.7)	165 (38.7)	56 (13.1)	28 (6.6)	8 (1.9)
Inadequate patient counselling contributes to inappropriate antibiotic use	145 (34.0)	172 (40.4)	58 (13.6)	41 (9.6)	10 (2.3)
Prescriber skills and knowledge affect antibiotic use	145 (34.0)	178 (41.8)	52 (12.2)	39 (9.2)	12 (2.8)
Patient self-medication influences antibiotic misuse	155 (36.4)	160 (37.6)	49 (11.5)	45 (10.6)	17 (4.0)
Inadequate supervision during medicine administration leads to inappropriate use	130 (30.5)	195 (45.8)	59 (13.8)	30 (7.0)	12 (2.8)
Antibiotics we use today could stop working properly in the future	126 (29.6)	160 (37.6)	70 (16.4)	57 (13.4)	13 (3.1)

### Awareness of antimicrobial resistance and stewardship

Most healthcare professionals correctly understood that antimicrobial resistance arises in bacteria, that many infections are becoming harder to treat, and that resistant infections can be very difficult or sometimes impossible to cure (Table 6). They also recognised that resistant bacteria can spread between people, inappropriate antibiotic use drives resistance, and this leads to more side‑effects and higher healthcare costs; awareness that resistance can complicate surgery, transplants and cancer care was high, although a minority still viewed it mainly as a distant or individual problem (Table 6).

**Table 6:** Item‑level awareness of antimicrobial resistance and antimicrobial stewardship among healthcare professionals.

Questions	Strongly agree n (%)	Agree n (%)	Neutral n (%)	Disagre n (%)	Strongly disagree n (%)
Antibiotic resistance occurs when bacteria, not humans, become resistant to antibiotics	161 (37.8)	152 (35.7)	48 (11.3)	53 (12.4)	12 (2.8)
Many infections are becoming increasingly resistant to antibiotics	143 (33.6)	200 (46.9)	47 (11.0)	28 (6.6)	8 (1.9)
If bacteria are resistant, it becomes very difficult or impossible to treat infections	153 (35.9)	175 (41.1)	52 (12.2)	39 (9.2)	7 (1.6)
Antibiotic resistance is a threat that could affect me or my family	112 (26.3)	154 (36.2)	70 (16.4)	74 (17.4)	16 (3.8)
Antibiotic resistance is only a problem in other countries, not here	61 (14.3)	83 (19.5)	56 (13.1)	147 (34.5)	79 (18.6)
Antibiotic resistance only affects people who take antibiotics frequently	87 (20.4)	157 (36.9)	66 (15.4)	88 (20.7)	28 (6.6)
Bacteria resistant to antibiotics can spread from person to person	84 (19.7)	131 (30.8)	61 (14.3)	106 (24.9)	44 (10.3)
Antibiotic-resistant infections could complicate surgeries, organ transplants, and cancer treatments	118 (27.7)	163 (38.3)	59 (13.8)	66 (15.5)	20 (4.7)
Inappropriate use of antibiotics increases antibiotic resistance	142 (33.3)	166 (39.0)	56 (13.1)	44 (10.3)	18 (4.2)
Inappropriate antibiotic use increases adverse effects and healthcare costs	134 (31.5)	173 (40.6)	57 (13.4)	47 (11.0)	15 (3.5)

### Factors associated with attitudes, knowledge, and awareness

In bivariate analyses, attitudes, knowledge and awareness varied significantly by age, education, years in practice, sex and profession (Table 7). Mean attitude and awareness scores rose with higher qualifications (from diploma/HND to master’s level) and were highest among participants older than 40 years, indicating more favourable profiles in the most experienced and highly educated groups (p < 0.05). Female participants and those with more than 10 years’ experience had higher mean knowledge and awareness scores than their counterparts, and bivariate differences were also observed across the collapsed professional categories (Table 7).

**Table 7:** Factors affecting Attitudes, Knowledge and awareness of the study sample.

	Attitudes	P-values	Knowledge	P-values	Awareness	P-values
**Age**		0.0142*		0.0000*		0.0016*
Less than 25 years	58.11 ± 7.93^A^		36.34 ± 6.55^R^		35.3 ± 5.41^A^	
25-30 years	59.61 ± 6.91		39.26 ± 6.66^A^		36.19 ± 4.88^A^	
31-40 years	57.48 ± 9.69		38.55 ± 7.91		36.93 ± 5.67	
More than 40 years	62.90 ± 6.83^A^		42.19 ± 6.16^A^		39.76 ± 5.76^R^	
**Sex**		0.6868		0.0174*		0.0080*
Male	58.80 ± 7.32		37.44 ± 6.78		35.50 ± 5.31	
Female	59.10 ± 8.30		39.05 ± 6.98		36.89 ± 5.22	
**Highest educational qualification**		0.0105*		0.0023*		0.0008*
Diploma/HND	54.92 ± 8.25^H^		35.46 ± 5.41		35.54 ± 6.58	
Bachelor’s degree	58.67 ± 7.67^H^		37.68 ± 6.82^H^		35.64 ± 5.11^H^	
Master’s degree	62.00 ± 6.81^R^		40.85 ± 6.59^H^		38.69 ± 5.24^H^	
P.H.D	59.07 ± 9.18		41.60 ± 8.10		38.40 ± 6.52	
**Years of practice**		0.5912		0.1469		0.0313*
Less than 1 year	58.5 ± 8.26		37.67 ± 6.89		36.36 ± 5.31	
1-5 years	59.19 ± 7.28		38.05 ± 6.76		35.71 ± 5.22^Y^	
6-10 years	58.11 ± 7.77		37.56 ± 6.88		35.65 ± 4.98	
More than 10 years	59.94 ± 9.18		40.79 ± 7.65		38.54 ± 6.05^Y^	
**Healthcare center**		0.1642		0.1044		0.4231
Demartino Hospital	59.68 ± 6.30		36.98 ± 5.97		36.58 ± 4.73	
Dr Sumait Hospital	60.95 ± 7.61		37.16 ± 7.96		35.39 ± 5.72	
Horyaal Hospital	59.41 ± 7.62		39.19 ± 6.08		35.28 ± 6.24	
Madina Hospital	58.08 ± 7.89		38.36 ± 7.06		36.06 ± 4.65	
Mogadishu Specialist Hospital	57.94 ± 8.42		39.88 ± 7.14		36.48 ± 5.12	
Shaafi Hospital	58.40 ± 8.41		37.35 ± 6.98		36.79 ± 5.55	
Welcare Hospital	56.50 ± 5.77		36.94 ± 6.33		34.88 ± 3.57	
Profession		0.0624		0.0000*		0.0014*
Others	55.94 ± 9.31		37.47 ± 7.34		36.21 ± 6.69	
Pharmacies	56.67 ± 7.49		35.58 ± 5.98^P^		33.67 ± 6.17	
Laboratory Technicians	58.59 ± 6.53		36.02 ± 7.16^R^		35.22 ± 5.27^P^	
Medical Doctors	60.43 ± 6.76		40.36 ± 6.35^P^		37.50 ± 4.86^P^	
Nurses/Midwives	58.36 ± 8.34		37.21 ± 6.86		35.38 ± 5.26	

* Significant (p < 0.05); A - Significant pair comparison of Age; H - Significant pair comparison of level of education; Y - Significant pair comparison of year of practice; P - Significant pair comparison of year of profession. P - Significant pair comparison reference group.

### Multivariable analysis

Holding a master’s degree remained an independent predictor of more positive attitudes toward actions to combat antimicrobial resistance: compared with diploma/HND holders, master’s graduates scored 6.33 points higher (95% CI 1.45-11.21; p = 0.011; Table 8). For general knowledge, age, years of practice, and workplace were significant; participants aged 25–30 years scored higher than those < 25 years (coefficient 2.97; 95% CI 1.38-4.56; p < 0.001), those with 6–10 years’ experience scored lower (-2.49; 95% CI -4.97-0.02; p = 0.049), and staff at Mogadishu Specialist Hospital had higher knowledge scores (2.52; 95% CI 0.21-4.84; p = 0.033; Table 8). For awareness, only 6–10 years of practice was independently associated, with scores 2.23 points lower than among those with <1 year of experience (95% CI -4.15-0.32; p = 0.023; Table 8). Although profession was significant in bivariate analyses, it was excluded from all three final multivariable models because it was not statistically significant in the full models, and models excluding it consistently showed lower AIC values. These model fit statistics indicated that the more parsimonious models without profession provided better fit.

**Table 8:** Multivariate analysis to assess the independent contribution of different factors affecting attitudes, knowledge, and awareness.

Predictors	Coefficient	Std. err.	t	P>|t|	95% Confidence Interval	
Attitudes on actions to combat antimicrobial resistance					Lower	Upper
**Constant**	55.93	2.29	24.45	0.000*	51.43	60.43
**Age group**						
25-30 years	1.48	0.84	1.76	0.080	0.17	3.13
31-40 years	-1.50	1.47	-1.02	0.308	1.39`	4.38
More than 40 years	4.03	2.08	1.94	0.053	0.06	8.12
**Highest educational qualification**						
Bachelor’s degree	3.05	2.16	1.42	0.158	1.19	7.29
Master’s degree	6.33	2.48	2.55	0.011*	1.45	11.21
P.H.D	2.15	3.07	0.70	0.483	3.88	8.19
**Healthcare center**						
Dr Sumait Hospital	1.24	1.34	0.93	0.355	1.40	3.88
Horyaal Hospital	-0.71	1.33	-0.54	0.592	1.90	3.32
Madina Hospital	-2.35	1.36	-1.72	0.085	0.33	5.03
Mogadishu Specialist Hospital	-1.88	1.33	-1.42	0.158	-4.49	0.73
Shaafi Hospital	-1.30	1.27	-1.02	0.306	1.19	3.79
Welcare Hospital	-3.15	2.02	-1.56	0.119	0.81	7.12
**General knowledge about antibiotics**						
**Constant**	34.34	2.07	16.61	0.000*	30.28	38.41
**Age group**						
25-30 years	2.97	0.81	3.67	0.000*	1.38	4.56
31-40 years	1.71	1.51	1.13	0.259	-1.27	4.69
More than 40 years	4.15	2.40	1.73	0.085	-0.58	8.87
**Sex**						
Female	0.26	0.76	0.34	0.734	-1.24	1.76
**Highest educational qualification**						
Bachelor’s degree	1.67	1.92	0.87	0.385	-2.10	5.43
Master’s degree	4.04	2.22	1.82	0.070	-0.33	8.40
P.H.D	3.03	2.76	1.1	0.272	-2.38	8.45
**Years of practice**						
1-5 years	-0.43	0.82	-0.53	0.597	-2.04	1.18
6-10 years	-2.49	1.26	-1.98	0.049*	-4.97	-0.02
More than 10 years	-0.41	1.96	-0.21	0.834	-4.26	3.44
**Healthcare center**						
Dr Sumait Hospital	0.09	1.19	0.08	0.938	-2.25	2.43
Horyaal Hospital	1.39	1.18	1.18	0.239	-0.93	3.71
Madina Hospital	0.21	1.22	0.17	0.862	-2.18	2.61
Mogadishu Specialist Hospital	2.52	1.18	2.14	0.033*	0.21	4.84
Shaafi Hospital	0.20	1.12	0.18	0.860	-2.01	2.41
Welcare Hospital	-0.41	1.80	-0.23	0.819	-3.96	3.13
**Awareness of antimicrobial resistance and stewardship**						
**Constant**	35.68	1.49	23.95	0.000*	32.75	38.61
**Age group**						
25-30 years	0.79	0.61	1.29	0.198	-0.41	1.99
31-40 years	0.60	1.16	0.51	0.609	-1.69	2.88
More than 40 years	2.57	1.85	1.39	0.164	-1.06	6.21
**Sex**						
Female	0.71	0.59	1.21	0.229	-0.45	1.86
**Highest educational qualification**						
Bachelor’s degree	0.18	1.48	0.12	0.903	-2.73	3.09
Master’s degree	2.82	1.72	1.64	0.102	-0.56	6.20
P.H.D	1.35	2.11	0.64	0.523	-2.79	5.49
**Years of practice**						
1-5 years	-1.07	0.64	-1.68	0.093	-2.32	0.18
6-10 years	-2.23	0.98	-2.29	0.023*	-4.15	-0.32
More than 10 years	-0.59	1.49	-0.39	0.695	-3.53	2.35

Model: Attitude; F *=* 2.65, p *=* 0.0020*, adjusted R^2^ *=* 4.4%, General knowledge; F *=* 2.69, p *=* 0.0004*, R2 *=* 6.0%, Awareness; F *=* 2.95, p *=* 0.0014*, adjusted R2 *=* 4.4%. *; Significant (p < 0.05).

Linear regression equations for significant predictors are attitude *=* 55.93 *+* 6.33 (master degree) *+* error; general knowledge *=* 34.34 *+* 2.97 (25–30 years) - 2.49 (6–10 years of experience) *+* 2.52 (Mogadishu Specialist Hospital) *+* error; and awareness *=* 35.68 - 2.23 (6–10 years of experience) *+* error.

## Discussion

This study provides a detailed assessment of healthcare professionals’ attitudes, knowledge, and awareness of antimicrobial resistance and stewardship in Mogadishu, Somalia, a protracted crisis setting characterized by weak regulation and fragmented healthcare delivery. Overall, the study found that healthcare professionals in Mogadishu hold broadly positive attitudes towards key actions to combat antimicrobial resistance, coupled with moderate awareness and only partly adequate factual knowledge about antibiotics. These findings point to a workforce that is conceptually aligned with stewardship principles but constrained by persistent misconceptions and uneven access to training and system support, a pattern widely reported among healthcare workers in African settings.

The high mean attitude score (78.6%) reflects widespread agreement that prescribing should be guided by clinical condition and microbiological results and that poor infection control, inappropriate prescribing, lack of diagnostics, self‑medication, and poor hygiene drive resistance.

This mirrors findings from Zambia and Egypt, where most respondents strongly endorsed stewardship‑aligned actions and recognized the role of prescribers’ skills, diagnostics, and infection prevention in shaping AMR [6,7].

In Mogadishu, such strong attitudinal support is striking given the fragile health system and weak regulation, which likely reflects growing global and regional visibility of AMR, as well as exposure to WHO messaging and national discussions around Somalia’s AMR action plan.

However, the same respondents reported variability in perceived overprescription and limited reference to formal guidelines, suggesting that positive attitudes coexist with structural constraints and inconsistent implementation. Large multi‑country African KAP surveys among pharmacy and other healthcare workers also report generally positive attitudes but document that guideline use, audit and feedback, and practical stewardship actions remain weak in everyday practice [26]. The situation in Mogadishu is plausibly more constrained, as recent work documenting irrational prescribing and high resistance levels in Somali hospitals highlights the combined effects of empirical treatment, over‑the‑counter availability, and weak institutional governance on prescribing behavior [11,27,28].

The mean knowledge score (69.3%) indicates that respondents understood some basic principles, for example, recognizing that frequent antibiotic use reduces treatment efficacy and that use should be strictly controlled, yet a substantial proportion endorsed incorrect statements about indications, treatment duration, and reuse of antibiotics. Many participants agreed, for instance, that antibiotics are used in the management of “all infections," that treatment can be stopped once a patient feels better (especially with expensive antibiotics), and that it is acceptable to reuse leftovers or buy the same antibiotic over the counter if previously helpful. These misconceptions are not unique to Somalia: studies in Ghana, Benin, and other African countries report similar confusion about antibiotic indications, stopping rules, and self‑medication, even among trained staff [29–32].

The coexistence of correct and incorrect beliefs likely reflects the interaction of pre‑service training that emphasizes basic pharmacology, on‑the‑job learning in resource‑constrained settings, and strong community norms around antibiotic use [33]. In many African contexts, including Somalia, high patient demand, limited diagnostic support, and easy access to antibiotics outside formal channels normalize practices that contradict stewardship principles, such as early discontinuation or self‑directed reuse of antibiotics [12]. Compared with the Zambian AMS‑team study, where 75% achieved “good” knowledge, helped by prior AMS training and structured programs, the Mogadishu scores are somewhat lower, which is expected given that most respondents here were not embedded in formal stewardship teams and work in a far less regulated system [6].

Awareness scores (72.1%) showed that most respondents correctly understood that resistance occurs in bacteria, not humans, that resistant infections are more difficult or sometimes impossible to treat, and that resistant organisms can spread between people. This aligns with African surveys reporting relatively good awareness of AMR as a biological and clinical phenomenon among healthcare workers, even where knowledge of specific stewardship interventions is weaker [29–31]. Many Mogadishu respondents also recognized that AMR can complicate surgeries, transplantation, and cancer care, suggesting an appreciation of its broader systems impact. Yet awareness of personal and local risk was more mixed: a notable minority perceived AMR mainly as a problem elsewhere or affecting only frequent antibiotic users, echoing findings from multi‑country African pharmacy surveys where participants recognized AMR as a global threat but were less convinced of its immediacy in their own practice.

In the Somali context, this misperception is particularly problematic, given evidence that resistance levels in common pathogens and the burden of AMR-related mortality are already very high [28,34]. The disconnect between global awareness and local risk perception may partly reflect limited feedback on local resistance patterns and the absence of routine surveillance and reporting systems that could make AMR more visible at the facility level.

With respect to participants’ socio‑demographic characteristics, this study identified important socio‑demographic gradients. Attitudes were higher among those with master’s degrees, and knowledge and awareness were higher among younger professionals and those working in some better-resourced facilities, while mid-career staff with 6–10 years of practice had significantly lower knowledge and awareness scores than both less-experienced and more-experienced colleagues. The positive association between advanced education and AMR‑related scores aligns with evidence from Ghana, Ethiopia, and multi‑country African KAP studies, where higher qualifications and specialist roles correlate with better understanding of AMR and stewardship [29,35]. The mid‑career “dip” contrasts with some reports that show the lowest scores among those with fewer years of experience, especially in community pharmacy settings. In Mogadishu, this pattern may have several explanations. First, younger staff, many within 1–5 years of practice, may have benefited from relatively recent training curricula that include more explicit coverage of AMR and stewardship, reflecting the growing prominence of these topics in global and regional policy [34]. Second, mid‑career professionals in this setting often shoulder heavy clinical workloads, multiple jobs across facilities, and managerial responsibilities, leaving limited time for continuing professional development on AMR or for engaging with emerging stewardship initiatives [11]. Third, more senior clinicians (>10 years) may have acquired additional exposure to complex cases, informal peer learning, and rare training opportunities, partially compensating for gaps in formal curricula [36,37]. In bivariate analyses, medical doctors showed higher knowledge and awareness scores than nurses/midwives, laboratory technicians, pharmacists, and other professions. However, after collapsing profession into five clinically meaningful categories (physicians, nurses/midwives, laboratory technicians, pharmacists, and others) to address sparse cell counts, profession was not independently significant in any of the three multivariable regression models and was excluded based on AIC model selection. This suggests that the apparent profession-level differences in KAA scores are largely explained by correlated factors such as educational level and years of experience, rather than representing an independent effect of professional cadre.

The relatively low adjusted R^2^ (≈4–6%) in the regression models indicates that demographic and professional factors explain only a small fraction of the variance in attitudes, knowledge, and awareness. This is consistent with African and global meta‑analyses showing that organizational culture, guideline availability, leadership support, supervision, AMR feedback, and patient pressures all shape AMR‑related behavior but are seldom captured in simple KAP surveys [26,34]. In Mogadishu, facility‑level differences in resources, access to microbiology, management priorities, and donor support likely contribute to differences between hospitals observed in the analysis, but disentangling these effects would require more detailed organizational data [11].

Overall, the Mogadishu findings are broadly in line with African KAP studies that report moderate knowledge, positive attitudes, and variable practice among healthcare workers, alongside strong correlations between these domains. The observed positive correlations between knowledge, awareness, and attitudes mirror results from multi‑country surveys showing that better knowledge tends to accompany more favorable attitudes and, where measured, better self‑reported practices [35,38]. Where the findings diverge is mainly in the balance between knowledge and attitudes and in the patterns by experience. The Zambian AMS‑team study and Egyptian hospital study reported higher overall knowledge and awareness among participants who were already embedded in stewardship structures and had access to AWaRe‑based guidelines and repeated training [6,7]. By contrast, the Mogadishu cohort comprises a broader mix of frontline staff in a system with minimal stewardship infrastructure, which plausibly explains the lower knowledge scores and the persistence of basic misconceptions.

Multi‑country African pharmacy studies highlight particularly poor KAP among unlicensed or informally trained dispensers; although the present study focuses on formally recognized facility‑based staff, the persistence of self‑medication‑friendly attitudes suggests that similar social and market pressures operate in Somalia [26,31]. Another difference is the context: Somalia’s health system has been deeply disrupted by protracted conflict, limited public financing and weak regulation, conditions that are more severe than those in many of the African countries represented in large KAP datasets. Recent Somali data show extreme resistance rates in common pathogens and high use of broad‑spectrum antibiotics, underscoring that prescribers operate in an environment where empirical treatment is often the only option and where trust in antibiotic efficacy is already eroding [3,4,39]. In such a setting, some of the “inappropriate” beliefs and behaviors captured in the knowledge items may be adaptive responses to resource constraints, patient expectations and the absence of accessible diagnostics, rather than simple deficits in understanding.

The combination of strong pro‑stewardship attitudes, mixed awareness and partial knowledge has direct implications for each of the five axes of Somalia’s National Action Plan on Combating Antimicrobial Resistance (NAP-AMR), which frames national AMR governance around awareness, surveillance, infection prevention and control, prudent antimicrobial use, and research and development, consistent with the WHO Global Action Plan on AMR [11,40].

Axis 1 emphasises awareness and education, using the identified gaps in knowledge about when to prescribe antibiotics, treatment duration, and self‑medication to define an evidence‑based in‑service training curriculum for Mogadishu health workers, prioritising mid‑career staff and lower‑scoring cadres and integrating AMR content into existing national continuing professional development systems. Axis 2 focuses on surveillance, suggesting that observed differences in facility knowledge scores can be used, together with microbiological resistance data, to identify hospitals where stewardship support should be prioritised, and underscoring the need to expand antibiogram capacity and link local data to GLASS so prescribers receive context‑specific resistance feedback. Axis 3 highlights infection prevention and control, noting that staff already recognise poor IPC as a key driver of resistance while compliance remains weak, and arguing that targeted IPC strengthening is a relatively low‑cost, high‑impact stewardship measure that fits the national IPC agenda and global evidence on reducing transmission and unnecessary empirical antibiotic use. Axis 4 addresses prudent use and regulation, pointing out that acceptance of self‑medication and over‑the‑counter reuse of antibiotics means facility‑based stewardship alone is insufficient, and supporting enforcement of essential medicines lists and treatment guidelines across sectors, combined with prescription audit and feedback and piloting multidisciplinary AMS teams in high‑volume hospitals as a practical entry point. Axis 5 covers research, indicating that the study contributes baseline information on AMR‑related knowledge, attitudes, and awareness across multiple facilities and cadres in Mogadishu, and that longitudinal follow‑up could help track change over time and assess the impact of stewardship interventions, providing useful evidence for national planning and for engaging international funders [7,12,31]. In fragile, urban settings such as Mogadishu, these interventions will only be effective if they are accompanied by broader policy measures to curb over‑the‑counter sales, strengthen regulation of private and informal providers, as well as improve infection prevention and control, which are all areas repeatedly flagged in Somali AMR reviews.

The findings of this study, therefore, provide both a baseline and a structured policy argument: each of the five NAP-AMR axes has a corresponding empirical finding from this study that can directly inform prioritisation, targeting, and monitoring of Somalia’s AMS response at the facility level.

## Study limitations

This pragmatic, facility-based study was conducted in seven hospitals in Mogadishu, so the findings may not represent healthcare workers in smaller, peripheral, rural, or informal facilities elsewhere in Somalia, and the self‑administered questionnaire is prone to social desirability bias, with no direct observation of actual prescribing or IPC practices. In addition, the use of consecutive convenience sampling among available staff may have introduced selection bias, and the predominantly young, early-career composition of the sample may limit the extent to which the findings reflect the perspectives of more experienced healthcare professionals. Some items were necessarily generic and may not fully capture hospital‑specific realities, and the cross‑sectional design cannot determine whether differences in attitudes, knowledge, and awareness are caused by education, experience, or workplace factors, or simply associated with them.

## Conclusions

Healthcare workers in major Mogadishu hospitals show strong pro‑stewardship attitudes and reasonably good awareness of antimicrobial resistance, but important knowledge gaps remain, particularly around indications, treatment duration, and self‑medication, with a notable mid‑career dip in scores. These patterns suggest that Somalia’s emerging AMR response should prioritise targeted, facility‑based stewardship interventions, especially for mid‑career staff and lower‑scoring cadres, alongside improvements in guidelines, diagnostics, surveillance, and antibiotic regulation to better align everyday prescribing and infection‑control practices with global AMR goals in a fragile health system.

## Supporting information

S1 File Questionnaire. (PDF)

S2 File Minimal dataset. (XLSX)
