# Supplementary material for: Antimicrobial resistance and stewardshipin clinical practice: A cross-sectional study of healthcare professionals in Mogadishu, Somalia
Source: PLOS Glob Public Health. 2026 Jun 18;6(6):e0006032. doi: 10.1371/journal.pgph.0006032 (PMC13278406; doi:10.1371/journal.pgph.0006032)
Supplement: S1 File — (PDF) [file pgph.0006032.s001.pdf]

Investigating Antimicrobial Usage Patterns and Awareness Among Health Workers in Mogadishu, Somalia.

This questionnaire is designed to gather information on antimicrobial usage patterns and awareness among health workers in Mogadishu/Banadir. Your responses will remain confidential and will be used solely for research purposes.

Thank you for your participation.

* Indicates required question

SECTION 1: Socio-Demographic Characteristics

1. Gender: *

   Mark only one oval.

   - ( ) Male
   - ( ) Female

2. Age (in years) *
   Da'daada

   ____

3. Highest education level *

Mark only one oval.

- Diploma or HND
- Bachelor’s Degree
- Master’s or Professional Degree
- Doctorate Degree
- Other: ____

4. Profession: *

Mark only one oval.

- Medical Doctor
- Nurse
- Midwife
- Pharmacist
- Laboratory Technician
- Radiologist
- Other: ____

5. Which Health Care Center do you work at? *

Mark only one oval.

- Dr Sumait Hospital
- Welcare Hospital
- Madina Hospital
- Mogadishu Specialist Hospital
- Shaafi Hospital
- Horyaal Hospital
- Demartino Public Hospital

6. Years of practice *

____

Section 2: How much do you agree with the following actions to combat antimicrobial resistance?

7. The patient's clinical condition influences the decision to start antimicrobial therapy. *

Mark only one oval.

- Strongly agree
- Agree
- Neutral
- Disagree
- Strongly Disagree

8. Some infections cannot be treated effectively with the current antibiotics available. *

Mark only one oval.

- Strongly agree
- Agree
- Neutral
- Disagree
- Strongly Disagree

9. There are policies and protocols guiding antibiotic use in this facility. *

Mark only one oval.

- Strongly agree
- Agree
- Neutral
- Disagree
- Strongly Disagree

10. All prescriptions are based on the hospital's protocol. *

Mark only one oval.

- Strongly agree
- Agree
- Neutral
- Disagree
- Strongly Disagree

11. Poor infection control practices cause the spread of antimicrobial resistance. *

Mark only one oval.

- Strongly agree
- Agree
- Neutral
- Disagree
- Strongly Disagree

12. Consulting with infectious disease experts helps control antimicrobial resistance. *

Mark only one oval.

- Strongly agree
- Agree
- Neutral
- Disagree
- Strongly Disagree

13. Obtaining local antibiotic resistance profiles helps improve antibiotic use. *

Mark only one oval.

- Strongly agree
- Agree
- Neutral
- Disagree
- Strongly Disagree

14. Targeting antimicrobial therapy to likely pathogens helps control resistance. *

Mark only one oval.

- Strongly agree
- Agree
- Neutral
- Disagree
- Strongly Disagree

15. Changing attitudes of prescribers and patients reduces unnecessary antibiotic use. *

Mark only one oval.

- Strongly agree
- Agree
- Neutral
- Disagree
- Strongly Disagree

16. Microbiological results in symptomatic patients influence the decision to start antibiotics. *

Mark only one oval.

- Strongly agree
- Agree
- Neutral
- Disagree
- Strongly disagree

17. Inappropriate prescribing habits contribute to antimicrobial resistance. *

Mark only one oval.

- Strongly agree
- Agree
- Neutral
- Disagree
- Strongly disagree

18. Lack of effective diagnostic tools contributes to inappropriate antibiotic use. *

Mark only one oval.

- Strongly agree
- Agree
- Neutral
- Disagree
- Strongly disagree

19. Patient self-medication with antibiotics contributes to antimicrobial resistance. *

Mark only one oval.

- Strongly agree
- Agree
- Neutral
- Disagree
- Strongly disagree

20. Poor hygiene practices in healthcare settings promote bacterial spread and resistance. *

Mark only one oval.

- Strongly agree
- Agree
- Neutral
- Disagree
- Strongly disagree

21. Antibiotics are overprescribed in this facility. *

Mark only one oval.

- Strongly agree
- Agree
- Neutral
- Disagree
- Strongly disagree

Section 3: General knowledge about antibiotics

22. Antibiotics are used in the management of all infections. *

Mark only one oval.

- Strongly agree
- Agree
- Neutral
- Disagree
- Strongly Disagree

23. Treatment with antibiotics should stop once a patient feels better, especially with expensive antibiotics. *

Mark only one oval.

- Strongly agree
- Agree
- Neutral
- Disagree
- Strongly Disagree

24. It is acceptable to use leftover antibiotics from a family member or friend if the symptoms are similar. *

Mark only one oval.

- Strongly agree
- Agree
- Neutral
- Disagree
- Strongly Disagree

25. It is acceptable to buy the same antibiotics without a prescription if they helped in the past. *

Mark only one oval.

- Strongly agree
- Agree
- Neutral
- Disagree
- Strongly Disagree

26. Frequent use of antibiotics may decrease treatment efficacy. *

Mark only one oval.

- Strongly agree
- Agree
- Neutral
- Disagree
- Strongly Disagree

27. Antibiotic use should be strictly controlled. *

Mark only one oval.

- Strongly agree
- Agree
- Neutral
- Disagree
- Strongly Disagree

28. Inadequate patient counseling contributes to inappropriate antibiotic use. *

Mark only one oval.

- Strongly agree
- Agree
- Neutral
- Disagree
- Strongly Disagree

29. Prescriber skills and knowledge affect antibiotic use. *

Mark only one oval.

- Strongly agree
- Agree
- Neutral
- Disagree
- Strongly Disagree

30. Patient self-medication influences antibiotic misuse. *

Mark only one oval.

- Strongly agree
- Agree
- Neutral
- Disagree
- Strongly Disagree

31. Inadequate supervision during medicine administration leads to inappropriate use. *

Mark only one oval.

- Strongly agree
- Agree
- Neutral
- Disagree
- Strongly Disagree

32. Antibiotics we use today could stop working properly in the future. *

Mark only one oval.

- Strongly agree
- Agree
- Neutral
- Disagree
- Strongly Disagree

Section 4: Awareness of antimicrobial resistance and stewardship

33. Antibiotic resistance occurs when bacteria, not humans, become resistant to antibiotics. *

Mark only one oval.

- Strongly agree
- Agree
- Neutral
- Disagree
- Strongly disagree

34. Many infections are becoming increasingly resistant to antibiotics. *

Mark only one oval.

- Strongly agree
- Agree
- Neutral
- Disagree
- Strongly disagree

35. If bacteria are resistant, it becomes very difficult or impossible to treat infections. *

Mark only one oval.

- Strongly agree
- Agree
- Neutral
- Disagree
- Strongly disagree

36. Antibiotic resistance is a threat that could affect me or my family. *

Mark only one oval.

- Strongly agree
- Agree
- Neutral
- Disagree
- Strongly disagree

37. Antibiotic resistance is only a problem in other countries, not here. *

Mark only one oval.

- Strongly agree
- Agree
- Neutral
- Disagree
- Strongly disagree

38. Antibiotic resistance only affects people who take antibiotics frequently. *

Mark only one oval.

- Strongly agree
- Agree
- Neutral
- Disagree
- Strongly disagree

39. Bacteria resistant to antibiotics can spread from person to person. *

Mark only one oval.

- Strongly agree
- Agree
- Neutral
- Disagree
- Strongly disagree

40. Antibiotic-resistant infections could complicate surgeries, organ transplants, and cancer treatments. *

Mark only one oval.

- Strongly agree
- Agree
- Neutral
- Disagree
- Strongly disagree

41. Inappropriate use of antibiotics increases antibiotic resistance. *

Mark only one oval.

- Strongly agree
- Agree
- Neutral
- Disagree
- Strongly disagree

42. Inappropriate antibiotic use increases adverse effects and healthcare costs. *

Mark only one oval.

- Strongly agree
- Agree
- Neutral
- Disagree
- Strongly disagree

Thank you for your participation

This content is neither created nor endorsed by Google.

Google Forms
