# Supplementary material for: Antimicrobial resistance and stewardshipin clinical practice: A cross-sectional study of healthcare professionals in Mogadishu, Somalia
Source: PLOS Glob Public Health. 2026 Jun 18;6(6):e0006032. doi: 10.1371/journal.pgph.0006032 (PMC13278406; doi:10.1371/journal.pgph.0006032)
Supplement: Attachment — Submitted filename: R1-Response to Reviewers.docx [file pgph.0006032.s004.docx]

**Response to Reviewers**

We thank the reviewers for their careful reading of our manuscript and for the constructive comments. We have revised the manuscript accordingly.

Our responses are provided below.

**Reviewer 1**

1. Study setting and sampling consistency

Comment:

There appears to be an inconsistency regarding the study setting and sampling framework. Line 25 indicates that the cross-sectional survey was conducted among 426 participants from 7 hospitals. However, Line 107 states that the study was implemented in 6 of the 20 districts, and a similar statement appears again on Line 130.

These descriptions create some ambiguity regarding the exact study scope. The authors are encouraged to clarify whether the study was conducted in 7 hospitals located within 6 districts, or if there is another explanation for this discrepancy. The study setting and sampling description should be harmonized across the manuscript to ensure consistency and clarity.

Response:

Thank you. We have clarified this point in the Methods section. The study was implemented in six districts, and data collection was ultimately conducted in seven hospitals located across those districts. We also clarified that the final sampling was facility-based.

Changes made: Methods, Study design and setting, lines 114-115; Study population and sample size, approximately lines 151-154.

2. Citation consistency for Kıratlı et al. (2024)

Comment:

There are inconsistencies in the way Kıratlı et al. (2024) is cited throughout the manuscript. Line 81 cites Kıratlı 2024b, line 345 cites Kıratlı et al. 2024a, line 433 also cites Kıratlı et al. 2024a. However, in the reference list (Line 582) only 2024a appears. If all these citations refer to the same publication, the in-text citations should be standardized accordingly. The reference should also follow exactly the format preferred by the authors, as indicated below:

Kıratlı K, Aysin M, Ali MA, Ali AM, Zeybek H, Köse Ş. Evaluation of Doctors’ Knowledge, Attitudes, Behaviors, Awareness and Practices on Rational Antimicrobial Stewardship in a Training and Research Hospital in Mogadishu, Somalia. Infect Drug Resist. 2024;17:2759-2771.

https://doi.org/10.2147/IDR.S463197

Response:

Thank you. Indeed its all same reference. We have standardized the in-text citation and reference entry throughout the manuscript and changed the reference style to the journal recommended style (Vancouver).

**Reviewer 2**

1. Selection bias and limitation to Mogadishu

Comment:

The study uses consecutive convenience sampling of available healthcare workers (mostly youth - selection bias) and sampling was limited to Mogadishu. The manuscript needs to recognize this limitation.

Response:

Thank you. We have strengthened the limitations section to acknowledge the use of consecutive convenience sampling, possible selection bias, the predominantly young and early-career sample, and limited generalizability beyond Mogadishu.

Changes made: Lines 507-515.

2. Theoretical relationship between knowledge, attitudes, and awareness

Comment:

The manuscript treats knowledge, attitudes, and awareness as separate domains but does not explain their relationship.

Response:

Thank you. We have retained the separate domains as measured by the questionnaire and further clarified their empirical relationships in the Results through correlation analysis and an added figure.

Changes made: Results, lines 252-266.

3. Reliability testing, construct validity, and pilot testing

Comment:

The survey was “adapted from a previous study”, but the manuscript does not report Cronbach's alpha, reliability testing, construct validity and pilot testing.

Response:

Thank you. We have added details on pre-testing, internal consistency from the pilot, Cronbach’s alpha for each study domain, and principal component analysis to support unidimensionality of the scales.

Changes made: Study population and sample size, lines 141-145; Data collection procedure, lines 182-187.

4. Justification for summing Likert-scale items into composite scores

Comment:

The study sums Likert-scale items into composite scores, and this must be justified using parameter analysis or ordinal regression models.

Response:

Thank you. We have now justified this approach by reporting internal consistency and PCA results, which supported treatment of the composite scores as continuous variables in subsequent analyses.

Changes made: Data collection procedure, lines 182-187.

5. Small profession categories

Comment:

Some professions have extremely small sample sizes, so it is better to collapse categories into broader groups, such as physicians, nurses/midwives, lab staff, pharmacists, and other professions. Interpenetration of knowledge score is also of concern.

Response:

Thank you. We collapsed profession into five broader categories: physicians, nurses/midwives, laboratory technicians, pharmacists, and other professions for descriptive and bivariate analyses.

Changes made: Data collection procedure, lines 191-194; Results, Characteristics of the study population, lines 225-228; Table 1 and Table 7.

6. Profession variable in multivariable regression

Comment:

Profession should be handled more appropriately in the analysis.

Response:

Thank you. After collapsing the profession categories, we re-analysed the variable. However, profession was not retained in the final multivariable models because it was not statistically significant and models excluding it had lower AIC values.

Changes made: Data analysis, lines 214-217 and following text in the same section.

7. Statistical reporting and model diagnostics

Comment:

Statistical reporting lacks confidence intervals, effect sizes, multicollinearity diagnostics and model diagnostics.

Response:

Thank you. We expanded the Data analysis section to report multicollinearity assessment using VIF, residual diagnostics, and model selection based on AIC.

Changes made: Data analysis, lines 209-214 and adjacent lines in the revised section.

8. Need for clearer policy relevance in Somalia

Comment:

The manuscript should clearly explain how findings inform antimicrobial stewardship policy in Somalia.

Response:

Thank you. We strengthened the discussion and conclusion to better link the findings to context-adapted AMS interventions, diagnostics, surveillance, regulation, and facility-level action in Somalia.

Changes made: Discussion and Conclusions, near the final section of the manuscript.

**Reviewer 3**

1. Abstract: please name the “other cadres”

Comment:

Please name the “other cadres.”

Response:

Thank you. We revised the Abstract to specify the broader participant categories more clearly.

Changes made: Abstract, Methods, lines 24-26.

2. Page 4, line 119: please state the “other clinicians”

Comment:

Please state the “other clinicians.”

Response:

Thank you. We revised the study population description to specify allied clinical staff more clearly.

Changes made: Study population and sample size, lines 119-121.

3. Sampling: “making a total of what?”

Comment:

Making a total of what?

Response:

Thank you. We revised this section to state clearly that the final sample size was 426 participants after extension of data collection to the remaining hospitals.

Changes made: Study population and sample size, lines 150.

4. Data collection procedure: order of questionnaire sections

Comment:

Shouldn’t “(ii)” come last?

Response:

Thank you. We revised the description of questionnaire sections for clarity and reordered the section description in the manuscript.

Changes made: Data collection procedure, lines 167-170.

5. Please include the data for diploma as well

Comment:

Please include the data for diploma as well.

Response:

Thank you. We have now explicitly included Diploma/HND in the Results narrative and retained it in Table 1.

Changes made: Results, Characteristics of the study population, line 225.
