# Supplementary material for: Antimicrobial resistance and stewardshipin clinical practice: A cross-sectional study of healthcare professionals in Mogadishu, Somalia
Source: PLOS Glob Public Health. 2026 Jun 18;6(6):e0006032. doi: 10.1371/journal.pgph.0006032 (PMC13278406; doi:10.1371/journal.pgph.0006032)
Supplement: Attachment — Submitted filename: R3-Response to reviewers comments.docx [file pgph.0006032.s005.docx]

**Response to editorial and reviewer comments**

**Editor comment**

1. Figure resubmission

We thank the editor for this guidance. We have reviewed all figure files using the PLOS NAAS tool and have included with this resubmission.

**Response to reviewer comments**

1. Do you mean purposively selected? If randomly selected, why are we talking about the fact that "they reflect a mix of public and private facilities that provide primary" care?

Response: We thank the reviewer for this observation. The districts were indeed randomly selected, and the original phrasing was misleading. The description of facility types was not a selection criterion but an outcome of the random selection process. We have revised the sentence to make this distinction clear (line 107).

2. Remove one of the double brackets on line 118.

Response: We thank the reviewer for catching this typographical error. The double bracket on line 118 has been corrected (line 118).
